# Factors associated with lymphedema therapist willingness to refer to surgery

**DOI:** 10.1007/s00520-025-10308-z

**Published:** 2026-01-16

**Authors:** Sarah Stolker, Kyle A. Pitzer, Judith M. Mwobobia, Karla T. Washington

**Affiliations:** 1 Washington University in St. Louis School of Public Health, St. Louis, MO USA; 2 Washington University in St. Louis School of Medicine, St. Louis, MO USA

**Keywords:** Lymphedema, Therapist, Practice, Surgery referral, Willingness

## Abstract

**Purpose:**

Treatment options for lymphedema have expanded in the last decade through the development of complex surgical interventions, whose innovations have transformed these options from a “last resort” to a potential way to restore lymphatic function, cosmesis, and reduce daily patient burden. Lymphedema therapists, who serve as a primary contact point for many individuals with lymphedema, may exhibit varying levels of acceptance of surgical intervention, potentially impacting patient education and access to these procedures. The current study seeks to identify factors associated with therapists’ willingness to refer patients with lymphedema for surgical consultation.

**Methods:**

Researchers conducted a cross-sectional exploratory study of 463 lymphedema therapists in the USA to determine factors associated with willingness to refer to surgery. Associations were examined between demographic variables, therapist characteristics (professional discipline, Lymphology Association of North America (LANA) certification, percent of caseload  with lymphedema, and self-reported research knowledge), practice factors (clinical setting, urbanicity, and number of practitioners at treatment site), and willingness to refer to surgery using bivariate and multivariate approaches.

**Results:**

Results identified significant associations between willingness to refer to surgery and practice setting, urbanicity, LANA certification, percent of caseload with lymphedema, and research knowledge. Specifically, willingness to refer was associated with urbanicity, LANA certification, over 50% lymphedema caseload, and therapists with higher self-reported research knowledge.

**Conclusions:**

Study results shed light on therapist and practice factors that may be influential in acceptance of innovations within lymphedema treatment, which could impact timeliness of referrals and quality of surgical outcomes.

## Introduction

Advances in surgery for the treatment of lymphedema have expanded the range of therapeutic options available for patients. Historically, conservative (non-surgical) interventions, such as Complete Decongestive Therapy (CDT), have been the standard of care for all stages of lymphedema, as early surgical options were associated with disfigurement, scarring, infection, and delayed wound healing [1]. In the last decade, innovations and advancements in surgical procedures, particularly microsurgical techniques, to address lymphedema have significantly challenged conservative therapy approaches. As evidence mounts, surgical innovations have the potential to shift existing lymphedema management paradigms [2]. This is particularly relevant to cancer patients, as cancer treatment is the leading cause of lymphedema in the USA and developed nations. Depending on the type of cancer and treatment received, lymphedema estimates can be as low as 0.7% for early stage endometrial cancer, to as high as 80% in malignant melanoma [3]. Breast cancer-related lymphedema, which affects approximately 1 in 5 survivors, is the most extensively studied form of lymphedema yet remains a persistent public health problem [4, 5].

Contemporary surgical techniques now focus on restoring lymphatic function by increasing drainage capacity [6–8]. Techniques like lymphaticovenular anastomosis (LVA) and vascularized lymph node transplant (VLNT) aim to reroute lymph fluid into the venous system or restore nodal function [8–11]. Innovations seek to reduce complications like donor-site lymphedema [12, 13], while reductive procedures like liposuction target fibroadipose tissue, especially when combined with compression therapy [14, 15]. Procedure selection requires identification of the predominant pathology (i.e., fluid dominant or fat dominant) with outcomes favoring earlier surgical intervention [16].

Patient identification for lymphatic surgery requires a multidisciplinary approach [17]. Limitations in lymphedema-specific knowledge among general healthcare providers put an increased reliance on lymphedema therapists for ongoing lymphedema patient management [18]. In a 2018 American Lymphedema Framework Survey, 96% of responding lymphedema therapists reported meeting training requirements set by the Lymphology Association of North America (LANA), which includes 135 h of training and 1 year of clinical practice in lymphedema management [19]. According to the International Society of Lymphology Consensus Document, lymphedema therapists’ specific training, education, and experience with the disease make them a crucial component to successful volume reduction and symptom management for patients with lymphedema [20].

In light of their expertise and role in coordinating care, as well as the timeline for optimal surgical outcomes, it is important to understand what factors influence lymphedema therapists’ attitudes towards lymphedema surgery. This study seeks to identify key professional and practice-related factors associated with willingness to refer lymphedema patients for surgical consultation. Additionally, we sought to identify self-reported barriers to surgical referral reported by therapists in various settings to shed light on ways surgeons can enhance educational efforts.

## Methods

### Design and setting

The survey was designed as a cross-sectional, exploratory study and was distributed to lymphedema therapists across the USA. A fully online format was chosen to maximize completion rates and to maintain data integrity and user-friendliness. The survey was active for 8 weeks from October 2024 to December 2024. REDCap (Research Electronic Data Capture), a secure, web-based data management platform, was used to create and administer the survey and manage the resulting data. The study was approved by Washington University in St. Louis Institutional Review Board (IRB ID 202409012).

### Participants and recruitment

Lymphedema therapists were eligible for study participation if they were over 18 years old, English speaking, and reported they were actively treating lymphedema patients within the USA. Exclusion criteria included therapists no longer practicing or those based outside of the USA. A total of 568 providers accessed the survey, of which 509 met inclusion criteria. Of the 509 eligible providers, *n* = 463 had valid data on our study outcome. After addressing missing data via list-wise deletion, analytic samples ranged from *n* = 420 to *n* = 443, depending on model variables.

The online survey invitation was distributed to a non-random sample of lymphedema therapists based in the USA, identified using alumni listservs from Klose Training, the Norton School of Lymphatic Therapy, and the Lymphedema Advocacy Group. The customized survey link was circulated by email and facilitated via snowball recruitment. We reached out to several lymphedema educators in the USA to reduce selection bias and improve the opportunity to capture a variety of therapists across the country. All participants provided informed consent.

### Measures and variables

With guidance from the Biostatistics, Epidemiology, and Research Design Core of the Washington University Institute of Clinical and Translational Sciences, survey development occurred through collaboration with key stakeholders, including lymphedema educators, clinicians, and individuals with lymphedema.

#### Willingness to refer

Our primary outcome was assessed via the following survey question: “Do you consider surgery to be an option for your patients with lymphedema?” Response options included “yes”, “no”, and “possibly but I need more information.” We conceptualized perceiving surgery as an option as a pragmatic proxy for willingness to refer because clinicians are unlikely to refer patients to interventions they do not view as appropriate, effective, or accessible for their patient population. For this analysis, we collapsed “no” and “possibly but I need more information” into a single category. This approach distinguished respondents who clearly endorsed a role for surgical intervention for patients from those expressing hesitancy or uncertainty due to perceived informational or practical barriers.

#### Demographics

Demographic variables included race and gender. Categories for race included White, Black, Asian, other, and prefer not to answer. The sample lacked racial diversity, and race was dichotomized into White versus non-White, including Black, Asian, and other races. While this approach oversimplifies subgroup differences, very small numbers in the non-White groups precluded other options. For gender, respondents were classified as female and male.

#### Practice setting

Practice setting variables included clinical setting, urbanicity, and number of practitioners at the treatment site. Categories for clinical setting included hospital/inpatient, outpatient ambulatory care, specialty practice/cancer center, and private practice. For urbanicity, respondents provided whether the location of their practice was urban, suburban, or rural. Lastly, participants were asked how many lymphedema practitioners were at their clinic (including themselves). Responses included 1, 2, 3, 4, or 5 +. For analysis, this variable was grouped into ≥ 3 versus < 3 therapists to capture whether working in a setting with more clinical peers might influence decision making.

Characteristics of interest in our analysis included professional discipline, LANA certification, percentage of caseload comprised of individuals with a lymphedema diagnosis, and research knowledge. Categories for professional discipline included physical therapist (PT), physical therapy assistant (PTA), occupational therapist (OT), occupational therapy assistant (OTA), massage therapist (MT), nurse (RN), nursing assistant, physician (MD/DO), and other. PT/PTA were combined into one category. OT/OTA were combined into another category. Remaining disciplines, including MT, were combined into “other.” LANA certification was self-reported certification (yes or no). Respondents were also asked how much of their patient caseload was comprised of individuals with lymphedema. Responses included 0%, < 25%, 26–50%, 51–75%, and > 75%. For the analysis, responses were grouped into two groups: ≤ 50% and > 50%. Finally, practitioner research knowledge was measured using the question, “do you consider yourself up to date on current lymphedema research,” and respondents could select one of 5 options: limited/no knowledge, mildly knowledgeable, moderately knowledgeable, very knowledgeable, completely knowledgeable. These responses were grouped into low/moderate and high research knowledge. We grouped the bottom three categories and the top two categories due to smaller cell size in the highest and lowest categories and our determination that the conceptually meaningful difference between research knowledge among therapists would likely be between those with moderate or lower and very knowledgeable or higher.

### Data analysis

Prior to modeling our data, we examined descriptive statistics for all our measures and variables. We used frequencies for all variables of interest. Additionally, we utilized chi-square tests to determine whether there were differences in our variables of interest by willingness to refer. To determine associations between our variables of interest and willingness to refer, we used logistic regression models with willingness to refer as the outcome and our variables of interest as the explanatory variables. We used a block-wise approach for this procedure. First, we estimated a model with only demographic characteristics (race and gender). Second, we added practice setting characteristics (practice setting, urbanicity, and number of therapists in practice). Lastly, we added therapist characteristics (professional discipline, LANA certification, % of caseload comprised of lymphedema patients, and research knowledge). We examined both estimates and significance values for individual variables and the model’s overall fit to the data. We also calculated odds ratios (OR) and their 95% confidence intervals (CI) for interpretation. We considered p values < 0.05 as statistical evidence of an association between predictors of interest and outcomes. Multicollinearity was examined using variance inflation factors (VIFs), and no evidence of multicollinearity between independent variables was found. Missing data were addressed using list-wise deletion.

## Findings

Demographically, most of our sample identified as White (89.4%) and female (94.0%), presented in Table 1. Most respondents practiced in outpatient ambulatory care settings (68.5%), followed by private practice (20.1%), specialty practice/cancer centers (5.4%), and hospitals/inpatient settings (5.0%). Respondents practiced in urban (27.0%), suburban (23.8%), and rural settings (48.6%) and most practice settings had fewer than 3 therapists (66%). Fifty percent of practitioners were in the field of physical therapy, 39% were in the field of occupational therapy, and 9% were in other fields. Nearly 75% did not have LANA certifications. About 55% reported more than 50% of their patients had lymphedema. Research knowledge was nearly evenly split, with 50.1% reporting low/moderate knowledge and 49.0% reporting high knowledge. Chi-square tests indicated that there was a statistically significant difference in willingness to refer to surgery by setting urbanicity, LANA certification, percent of lymphedema patients seen, and research knowledge. Specifically, there was a greater proportion of practitioners in urban and rural settings and a lower proportion of practitioners in suburban settings willing to refer compared to not, and a greater proportion of practitioners with LANA certifications, who saw greater than 50% of lymphedema patients in their caseload and who reported high research knowledge, were willing to refer to surgery than not.

**Table 1:** Summary of participant demographics and characteristics by willingness to refer for surgery^2^

	Overall (*N* = 463)	No (*N* = 293)	Yes (*N* = 170)	*p*-value^1^
**Race**				
White	414 (89.4%)	262 (89.4%)	152 (89.4%)	0.941
Non-white	32 (6.9%)	21 (7.2%)	11 (6.5%)	
Missing	17 (3.7%)	10 (3.4%)	7 (4.1%)	
**Gender**				
Female	435 (94.0%)	274 (93.5%)	161 (94.7%)	0.884
Male	24 (5.2%)	16 (5.5%)	8 (4.7%)	
Missing	4 (0.9%)	3 (1.0%)	1 (0.6%)	
**Practice Setting**				
Outpatient Ambulatory Care	317 (68.5%)	195 (66.6%)	122 (71.8%)	0.075
Hospital/Inpatient	23 (5.0%)	18 (6.1%)	5 (2.9%)	
Specialty Practice/Cancer Center	25 (5.4%)	12 (4.1%)	13 (7.6%)	
Private Practice	93 (20.1%)	65 (22.2%)	28 (16.5%)	
Missing	5 (1.1%)	3 (1.0%)	2 (1.2%)	
**Urbanicity**				
Urban	125 (27.0%)	67 (22.9%)	58 (34.1%)	< 0.001
Suburban	110 (23.8%)	90 (30.7%)	20 (11.8%)	
Rural	225 (48.6%)	135 (46.1%)	90 (52.9%)	
Missing	3 (0.6%)	1 (0.3%)	2 (1.2%)	
**More than 3 Therapists**				
No	306 (66.1%)	202 (68.9%)	104 (61.2%)	0.079
Yes	154 (33.3%)	88 (30.0%)	66 (38.8%)	
Missing	3 (0.6%)	3 (1.0%)	0 (0%)	
**Professional Discipline**				
PT	231 (49.9%)	136 (46.4%)	95 (55.9%)	0.103
OT	180 (38.9%)	120 (41.0%)	60 (35.3%)	
Other	41 (8.9%)	30 (10.2%)	11 (6.5%)	
Missing	11 (2.4%)	7 (2.4%)	4 (2.4%)	
**LANA Certification**				
No	339 (73.2%)	244 (83.3%)	95 (55.9%)	< 0.001
Yes	124 (26.8%)	49 (16.7%)	75 (44.1%)	
**Patients with Lymphedema Seen**				
≤ 50	208 (44.9%)	152 (51.9%)	56 (32.9%)	< 0.001
> 50	253 (54.6%)	141 (48.1%)	112 (65.9%)	
Missing	2 (0.4%)	0 (0%)	2 (1.2%)	
**Research Knowledge**				
Low/Moderate	232 (50.1%)	169 (57.7%)	63 (37.1%)	< 0.001
High	227 (49.0%)	123 (42.0%)	104 (61.2%)	
Missing	4 (0.9%)	1 (0.3%)	3 (1.8%)	

^1^*P* values were derived from Chi-square tests

^2^*PT* = Physical therapist, *OT* = Occupational Therapist, *LANA* = Lymphology Association of North America

Our final logistic model was largely consistent with the bivariate results, except for urbanicity and lymphedema caseload (Table 2). Our results indicated an association between willingness to refer and urbanicity of setting, LANA certification, and research knowledge. When exponentiated to odds ratios (Table 3), our results indicated that practitioners in suburban settings had 70% lesser odds of being willing to refer patients to surgery (OR = 0.30, CI = [0.15, 0.61], p < 0.001) compared to those in urban settings. In contrast, those with a LANA certification had 2.88 times greater odds of being willing to refer (OR = 2.88, CI = [1.75, 4.76]) compared to those without, and those reporting high research knowledge had 87% greater odds of being willing to refer (OR = 1.87, CI = [1.18, 2.96]) compared to those reporting low/moderate research knowledge. The model also showed rural settings had lesser odds of willingness to refer compared to urban, while providers who had a lymphedema caseload greater than 50% had greater odds of willingness to refer compared to those with less than 50%; however, these results were not statistically significant.

**Table 2:** Logistic model results of demographics, practice characteristics, therapist characteristics, and willingness to refer for surgery^1,2^

	Demographics Only		Demographics + Practice Characteristics		Demographics + Practice and Therapist Characteristics
Race (ref = White)					
Non-White	−0.087		−0.209		0.114
	(0.388)		(0.400)		(0.425)
Gender (ref = Female)					
Male	−0.212		−0.235		−0.308
	(0.471)		(0.495)		(0.515)
Setting (ref = Outpatient Care)					
Hospital/Inpatient			−0.728		−0.500
			(0.544)		(0.579)
Specialty Practice/Cancer Center			0.369		0.302
			(0.432)		(0.466)
Private Practice			−0.412		−0.425
			(0.285)		(0.333)
Urbanicity (ref = Urban)					
Suburban			−1.388 ***		−1.195 ***
			(0.328)		(0.361)
Rural			−0.279		−0.293
			(0.240)		(0.259)
Therapists in Practice (ref = Less than 3)					
3 + Therapists			0.156		0.001
			(0.227)		(0.247)
Professional Discipline (ref = PT)					
OT					−0.077
					(0.244)
Other					0.167
					(0.469)
LANA Certification (ref = No)					
Yes					1.057 ***
					(0.254)
Lymphedema Population Seen (ref ≤ 50%)					
> 50%					0.274
					(0.251)
Research Knowledge (ref = Low/Moderate)					
High					0.624 **
					(0.235)
N. obs	443		433		420
Log Likelihood	−290.743		−269.000		−241.682
AIC	587.487		556.000		511.363

^1^*** *p* < 0.001; ** *p* < 0.01; * *p* < 0.05

^2^*PT* = Physical therapist, *OT* = Occupational Therapist, *LANA* = Lymphology Association of North America

**Table 3:** Odds ratios and confidence intervals for willingness to refer for surgery^1^

	Demographics Only		Demographics + Practice Characteristics		Demographics + Practice and Therapist Characteristics	
Variable	OR	CI 95%	OR	CI 95%	OR	CI 95%
Race (ref = White)						
Non-White	0.92	[0.41, 1.93]	0.81	[0.36, 1.75]	1.12	[0.47, 2.54]
Gender (ref = Female)						
Male	0.81	[0.3, 1.97]	0.79	[0.28, 2.02]	0.74	[0.25, 1.96]
Setting (ref = Outpatient Care)						
Hospital/Inpatient			0.48	[0.15, 1.32]	0.61	[0.18, 1.78]
Specialty Practice/Cancer Center			1.45	[0.62, 3.41]	1.35	[0.54, 3.41]
Private Practice			0.66	[0.37, 1.15]	0.65	[0.34, 1.24]
Urbanicity (ref = Urban)						
Suburban			0.25	[0.13, 0.47]	0.30	[0.15, 0.61]
Rural			0.76	[0.47, 1.21]	0.75	[0.45, 1.24]
Therapists in Practice (ref = Less than 3)						
3 + Therapists			1.17	[0.75, 1.82]	1.00	[0.61, 1.62]
Professional Discipline (ref = PT)						
OT					0.93	[0.57, 1.5]
Other					1.18	[0.46, 2.94]
LANA Certification (ref = No)						
Yes					2.88	[1.75, 4.76]
Lymphedema Population Seen (ref = ≤ 50%)						
> 50%					1.32	[0.8, 2.16]
Research Knowledge (ref = Low/Moderate)						
High					1.87	[1.18, 2.96]

^1^*PT* = Physical therapist, *OT* = Occupational Therapist, *LANA* = Lymphology Association of North America

Our final model correctly predicted about 70% of cases, indicating an acceptable fit to the data.

## Conclusions/interpretation

This study aimed to determine factors associated with lymphedema therapists’ willingness to refer patients for lymphedema surgery. Observed associations can be a valuable foundation for identifying research questions and informing hypothesis-driven work; however, they do not constitute evidence of causality [21]. We identified positive significant associations between specific therapist- and practice-related factors and willingness to refer. These include setting, urbanicity, percentage of caseload comprised of patients with lymphedema, LANA certification, and higher self-reported research knowledge. We found no significant associations between willingness to refer and therapist race, gender, practice setting, and hours worked per week.

### Urbanicity

Our study’s findings indicate that, compared to practitioners in urban settings, practitioners in suburban settings had 70% lesser odds of being willing to refer patients to surgery. The findings regarding suburban willingness to refer are somewhat surprising. However, suburban rehabilitation practices may have fewer institutional pathways for surgical referral than urban practices, limiting their knowledge and endorsement of surgery as a potentially beneficial option for patients with lymphedema. This unexpected finding suggests that further research on suburban rehabilitation practices is needed. For rural settings, although not statistically significant, practitioners in rural settings had 25% lesser odds of being willing to refer patients to surgery than their urban counterparts. Evidence on the unique challenges of rural rehabilitation settings, such as larger caseloads, limited referral options, decreased access to resources, and limited access to continuing education, could help explain these findings [21, 22].

### Lymphedema-specific caseload

A study comparing referral patterns of recent graduate and experienced physical therapists found that referral decisions were influenced by clinicians’ perceived ability to recognize clinical patterns, with more experienced therapists referring to a broader range of practitioners and reporting more confidence in these decisions [23]. Although our survey did not include a direct measure of years of experience, bivariate analyses indicate that therapists with caseloads comprising more than 50% lymphedema patients were more likely to be willing to refer. This directional association was also observed in the multivariate model, although it was not statistically significant.

### LANA certification

LANA certification in our sample was 27%, slightly lower than the 33% reported by the American Lymphedema Framework Project (ALFP) in 2021, which also noted that LANA-certified therapists’ high clinical knowledge correlates with increased and appropriate referral for lymphedema treatment and surgery [19]. LANA certification originated in 1998 in response to American Cancer Society recommendations for quality control in lymphedema care [23]. Educational gaps have been identified with regard to lymphatic examination and intervention skills in physical therapy programs [24]. Thus, graduates are encouraged to utilize lymphedema professionals or pursue training after graduation. LANA’s 2020 State of the Industry Survey described variability in practice patterns of lymphedema therapists, acknowledging the autonomy and individuality of treatment approaches within the field of lymphedema therapy [25].

### Self-reported knowledge

Our study’s findings also demonstrate a significant relationship between research knowledge and willingness to refer to surgery. These findings are echoed in the lymphedema and rehabilitation literature demonstrating that clinical knowledge is associated with increased referral [18, 26]. A large-scale survey of physicians at Kaiser Permanente found that clinicians with higher clinical knowledge on the risks of breast cancer-related lymphedema were more likely to make referrals for lymphedema, showing that clinician education is warranted and impactful [27]. Although the current study did not include potentially relevant variables such as years of experience, both studies support the broader notion that clinical education may improve referral practices. A study measuring lymphedema knowledge among oncology nurse navigators demonstrated that higher clinical knowledge and expertise in lymphedema among this group of clinicians was associated with increased likelihood of educating patients about risk factors, thus contributing to early identification and prompt treatment of lymphedema [28].

### Willingness to refer

According to Everett Roger’s Diffusion of Innovations Theory, five key characteristics influence whether and how innovations are adopted: relative advantage, compatibility, complexity, trialability, and observability [29]. Drawing on comprehensive work applying this model to the diffusion of innovations in health care, we look to the attributes of the innovation, microsurgical procedures, to help explain our findings regarding the variability in willingness to refer [30, 31]. Modern microsurgical techniques may offer what is described as a *relative advantage* over conservative treatment due to their ability to restore lymphatic function for select patients; however, these benefits may not be fully recognized or remain ambiguous across the lymphedema therapist community [30]. *Compatibility*, or how consistent a new practice aligns with existing values and norms, may be challenged by the diversity in the lymphedema therapist workforce. Because lymphedema therapists come from a range of rehabilitation backgrounds, variation may exist in how surgical innovation is perceived or integrated into practice, especially when challenging longstanding skepticism toward surgical approaches [29]. *Complexity* may be another relevant factor since sophisticated and highly technical innovations, such as microsurgical techniques, are often associated with slower adoption [30]. *Trialability* and *observability*—the ability to test and directly witness the outcomes of an innovation—could be limited in certain settings. For example, suburban therapists may have fewer opportunities to observe successful surgical outcomes within their patient populations or collaborate with surgeons. Without firsthand exposure to positive results, therapists may be less willing to endorse surgery to their patients with lymphedema. Finally, the lack of consistent insurance coverage for surgical procedures may reduce the perceived relative advantage of these options for both therapists and their patients, further hindering adoption.

### Implications for practice

Lymphedema therapists are often the most knowledgeable point of contact for individuals with lymphedema. Despite the recent advent of surgical techniques that expand the options for patients with lymphedema, some lymphedema therapists may not consider surgery as an option for their patients. While some patients may not be optimal surgical candidates, an overarching unwillingness to refer for surgical consultation could discourage surgical referrals, thereby limiting a patient’s options, simply based on therapist or practice characteristics. Through identifying factors associated with therapist willingness to refer for surgical consultation, we seek to highlight potential disparities in treatment approaches that may alter the timing of surgical consultation and, potentially, surgical outcomes for lymphedema.

### Study limitations

Survey distribution was limited to two longstanding lymphedema training programs and the Lymphedema Advocacy Group, which may limit generalizability to therapists trained elsewhere or less involved in advocacy. The inability to calculate a response rate due to the distribution method further limits our understanding of potential non-response bias and representativeness of our sample.The sample also lacked racial diversity, precluding meaningful subgroup analyses. The innovative nature of this study limited access to data on which to base an a priori power analysis. Thus, it is possible that analysis of a larger sample might have identified additional statistically significant variables. Additionally, the cross-sectional design of the study precludes any causal inferences regarding the observed associations.

In addition to design and sampling considerations, the study is also limited by the variables not included in the survey, such as years of experience, which may be an important indicator of clinical expertise and could influence referral behaviors, or on lymphedema-specific patient population served, which could provide a more nuanced understanding of referral decisions. Importantly, the survey did not capture providers’ rationale for unwillingness to refer to surgery, limiting insight into modifiable barriers and targeted educational efforts. Furthermore, several ordinal variables (e.g., number of therapists, percentage of patients with lymphedema, and self-reported research knowledge) were dichotomized according to conceptually meaningful categories. This approach may have reduced the statistical power or obscured variability in the data. Finally, self-reported research knowledge is subjective and may not accurately reflect actual competency.

## Data Availability

Due to the ongoing nature of the research, the data are not publicly available at this time but may be made available upon reasonable request to the authors.
